# Supplementary material for: Estimating the mortality burden of large scale mining projects—Evidence from a prospective mortality surveillance study in Tanzania
Source: PLOS Glob Public Health. 2021 Oct 13;1(10):e0000008. doi: 10.1371/journal.pgph.0000008 (PMC10021452; doi:10.1371/journal.pgph.0000008)
Supplement: S1 Appendix — (DOCX) [file pgph.0000008.s001.docx]

# S1 Appendix: WHO VA target list of causes of death list

**Cause of death list for VA with corresponding ICD-10 codes.**

Column 1 contains the code for the VA entity. Column 2 lists the related titles. Column 3 lists the ICD-10 codes that would be used if the condition labelled by column 2 were coded to ICD-10. The third column lists the ICD-10 codes that relate to the text label of the cause of death category in Column 2.

| **Verbal autopsy code** | **Verbal autopsy title** | **ICD-10**  **codes (from ICD - 2016)** |
| --- | --- | --- |
| **VAs-01 Infectious and parasitic diseases** | |  |
| VAs-01.01 | Sepsis | A40-A41 |
| VAs-01.02 | Acute respiratory infection, including pneumonia | J00-J22; J85 |
| VAs-01.03 | HIV/AIDS related death | B20-B24 |
| VAs-01.04 | Diarrheal diseases | A00-A09 |
| VAs-01.05 | Malaria | B50-B54 |
| VAs-01.06 | Measles | B05 |
| VAs-01.07 | Meningitis and encephalitis | A39; G00- G05 |
| VAs-01.08 | Tetanus[1] | A33-A35 |
| VAs-01.09 | Pulmonary tuberculosis | A15-A16 |
| VAs-01.10 | Pertussis | A37 |
| VAs-01.11 | Haemorrhagic fever[2]        Ex | A92-A96, A98-A99 |
| VAs-01.12 | Dengue fever | A97 |
| VAs-01.13 | Coronavirus disease (COVID-19) | U07.1; U07.2 |
| VAs-01.99 | Unspecified infectious disease | A17-A19; A20-  A32;  A36; A38; A42A89; B00-B04;  B06-B19;  B25-B49; B55-B99 |

# Non-communicable diseases

| VAs-  98 | Other and unspecified non- communicable disease    **Note:**  **This group covers all non-communicable conditions that could not be assigned to another category in this section. There is a separate category for cases where the cause of death is unknown.** | D65-D89; E00-E07; E15E35; E50-E90; F00-F99; G06-G09; G10-G37; G50G99; H00-H95; J30-J39;  J47-J84; J86-J99; K00K31; K35-K38 K40-K69;  K77-K93 L00-L99; M00M99; N00-N16; N20-N99; | |
| --- | --- | --- | --- |
| **VAs-02 Neoplasms** | | | |
| VAs-  02.01 | Oral neoplasms | C00-C06 | |
| VAs-  02.02 | Digestive neoplasms | C15-C26 | |
| VAs-  02.03 | Respiratory neoplasms | C30-C39 | |
| VAs-  02.04 | Breast neoplasms | C50 | |
| VAs-  02.05 | Female reproductive neoplasms | C51-C58 | |
| VAs-  02.06 | Male reproductive neoplasms | C60-C63 | |
| VAs02.99 | Other and unspecified neoplasms | C07-C14;  C64-D48;  C91-C95 | C40-C49; |

| **VAs-03 Nutritional and endocrine disorders** | |  |
| --- | --- | --- |
| VAs-  03.01 | Severe anaemia | D50-D64 |
| VAs-  03.02 | Severe malnutrition | E40-E46 |
| VAs-  03.03 | Diabetes mellitus | E10-E14 |
| **VAs-04 Diseases of the circulatory system** | |  |
| VAs-  04.01 | Acute cardiac disease[3] | I11.0; I20-I26; I46.1; I46.9; I50.1 |
| VAs-  04.02 | Stroke | I60-I69 |
| VAs-  04.03 | Sickle cell with crisis | D57 |
| VAs04.99 | Other and unspecified  cardiac disease | I00-I10; I11.9-I15; I27-I46.0; I47-I50.0; I50.9-I52; I70-I99 |
| **VAs-05 Respiratory disorders** | |  |
| VAs-  05.01 | Chronic obstructive  pulmonary disease (COPD) | J40-J44 |
| VAs-  05.02 | Asthma | J45-J46 |
| **VAs-06 Gastrointestinal disorders** | |  |
| VAs-  06.01 | Acute abdomen | R10 |
| VAs-  06.02 | Liver cirrhosis[4] | K70.2; K70.3; K71.7; K74 |
| **VAs-07 Renal disorders** | |  |
| VAs-  07.01 | Renal failure | N17-N19 |
| **VAs-08 Mental and nervous system disorders** | |  |
| VAs-  08.01 | Epilepsy | G40-G41 |

| **VAs-09 Pregnancy-, childbirth and puerperium-related disorders** | | |
| --- | --- | --- |
| VAs-  09.01 | Ectopic pregnancy | O00 |

| VAs-  09.02 | Abortion-related death | O03-O08 |  |
| --- | --- | --- | --- |
| VAs09.03 | Pregnancy-induced hypertension | O10-O16 |  |
| VAs09.04 | Obstetric haemorrhage | O46; O67; O72 |  |
| VAs-  09.05 | Obstructed labour | O63-O66 |  |
| VAs09.06 | Pregnancy-related sepsis | O75.3; O85 |  |
| VAs-  09.07 | Anaemia of pregnancy | O99.0 |  |
| VAs-  09.08 | Ruptured uterus | O71.0-O71.1 |  |
| VAs09.99 | Other and unspecified maternal cause | O01-O02; O20-O45;  O62; O68-O70;  O71.3-O71.9;  O73-O84; O86-O99 | O47- |
| **VAs-10 Neonatal causes of death** | | |  |
| VAs-  10.01 | Prematurity or low birth weight | P05; P07 |  |
| VAs-  10.02 | Birth asphyxia[5] | P20-P22 | |
| VAs-  10.03 | Neonatal pneumonia | P23-P24 | |
| VAs-  10.04 | Neonatal sepsis | P36 | |
| VAs-  10.05 | Neonatal tetanus | A33 | |
| VAs-  10.06 | Congenital malformation | Q00-Q99 | |
| VAs10.99 | Other and unspecified perinatal  cause of death | P00-P04; P08-P15; P25-P35; P37-P94; P96 | |

| **VAs-11 Stillbirths** | | |
| --- | --- | --- |
| VAs-  11.01 | Fresh stillbirth | P95 |
| VAs-  11.02 | Macerated stillbirth | P95 |
| **VAs-12 External causes of death** **Note:**  **The list of questions contains sub questions that allow for more specificity for accidents.** | | |
| VAs-  12.01 | Road traffic accident | [6] |
| VAs-  12.02 | Other transport accident |  |
| VAs-  12.03 | Accidental fall | W00-W19 |
| VAs-  12.04 | Accidental drowning and submersion | W65-W74 |
| VAs-  12.05 | Accidental exposure to smoke, fire and flames | X00-X19 |
| VAs-  12.06 | Contact with venomous animals and plants | X20-X29 |
| VAs12.07 | Accidental poisoning and exposure to noxious substance | X40-X49 |
| VAs-  12.08 | Intentional self-harm | X60-X84; Y87.0 |
| VAs-  12.09 | Assault | X85-Y09; Y87.1 |
| VAs-  12.10 | Exposure to force of nature | X30-X39 |
| VAs12.99 | Other and unspecified external cause of death | (S00-T99); W20-W64; W75-  W99;  X10-X19; X50-X59; Y10-  Y84; Y86; Y87.2; Y88-Y89; |
|  |  |  |
| VAs-99 | Cause of death unknown | R95-R99 |

1. Excludes: Neonatal tetanus VAs-10.05
2. Excludes: Dengue VAs-01.12
3. Includes: Ischaemic heart disease; Pulmonary embolism; Sudden cardiac death; Cardiac arrest, unspecified; Left ventricular failure; and Hypertensive heart disease with heart failure [4] Includes Alcoholic fibrosis/ cirrhosis; Toxic liver cirrhosis; Fibrosis and cirrhosis of liver,

excluding alcoholic and toxic, but including ‘unspecified liver cirrhosis’ [5] Includes: Hypoxia and respiratory distress

[6] Distinction on the codes between VAs-12.01 and VAs 12.02 is on the basis whether the death was a road traffic accident. V01.1;V02.1;V03.1;V04.1;V05.1;V06.1; V09.2;V09.3; V10.4-V10.9; V11.4-V11.9; V12.4V12.9; V13.4-V13.9; V14.4-V14.9; V15.4-V15.9; V16.4-V16.9; V17.4-V17.9; V18.4-V18.9; V19.4-V19.9;

V20.4-V20.9; V21.4-V21.9; V22.4-V22.9; V23.4-V23.9; V24.4-V24.9; V25.4-V25.9; V26.4-V26.9; V27.4V27.9; V28.4-V28.9; V29.4-V29.9; V30.5-V30.9; V31.5-V31.9; V32.5-V32.9; V33.5-V33.9; V34.5-V34.9;

V35.5-V35.9; V36.5-V36.9; V37.5-V37.9; V38.5-V38.9; V39.4-V39.9; V40.5-V40.9; V41.5-V41.9; V42.5V42.9; V43.5-V43.9; V44.5-V44.9; V45.5-V45.9; V46.5-V46.9; V47.5-V47.9; V48.5-V48.9; V49.4-V49.9;

V50.5-V50.9; V51.5-V51.9; V52.5-V52.9; V53.5-V53.9; V54.5-V54.9; V55.5-V55.9; V56.5-V56.9; V57.5V57.9; V58.5-V58.9; V59.4-V59.9; V60.5-V60.9; V61.5-V61.9; V62.5-V62.9; V63.5-V63.9; V64.5-V64.9;

V65.5-V65.9; V66.5-V66.9; V67.5-V67.9; V68.5-V68.9; V69.4-V69.9; V70.5-V70.9; V71.5-V71.9; V72.5V72.9; V73.5-V73.9; V74.5-V74.9; V75.5-V75.9; V76.5-V76.9; V77.5-V77.9; V78.5-V78.9; V79.4-V79.9;

V80.0-V80.9;V81.1-V81.9; V82.1-V82.9; V83.0-V83.3; V84.0-V84.3; V85.0-V85.3; V86.0-V86.3; V87.0V87.9; V89.2-V89.3; Y85.0; V90-V99; Y85.9
