# Supplementary material for: Estimating the mortality burden of large scale mining projects—Evidence from a prospective mortality surveillance study in Tanzania
Source: PLOS Glob Public Health. 2021 Oct 13;1(10):e0000008. doi: 10.1371/journal.pgph.0000008 (PMC10021452; doi:10.1371/journal.pgph.0000008)
Supplement: Attachment — Submitted filename: response_to_reviewers.docx [file pgph.0000008.s002.docx]

**Manuscript Number:** PGPH-D-21-00132 **Manuscript Title:** Estimating the mortality burden of large scale mining projects – Evidence from a prospective mortality surveillance study in Tanzania

**Cover letter**

Dar-es-Salaam, 7 July 2021

Dear Dr. Chirico

Thank you for your kind email from July 2^nd^, 2021. We were pleased to note the overall positive appraisal of our research and grateful for your invitation to revise and resubmit our manuscript.

We have carefully revised our manuscript in light of the reviewer’s feedback. Please find our point-by-point responses attached below. To further assist in the readily reviewing our changes, we indicate the line numbers where the revised manuscript has changed in our reply. In the revised manuscript, all changes are highlighted in yellow.

We look forward to hearing from you soon – please let us know if you have any questions or require any further information.

Yours sincerely,

Isaac Lyatuu (on behalf of all the co-authors)

**Point by point responses to reviewers**

**Manuscript Number:** PGPH-D-21-00132 **Manuscript Title:** Estimating the mortality burden of large-scale mining projects – Evidence from a prospective mortality surveillance study in Tanzania

1. Does this manuscript meet PLOS Global Public Health's [publication criteria](https://journals.plos.org/climate/s/criteria-for-publication)? Is the manuscript technically sound, and do the data support the conclusions? The manuscript must describe methodologically and ethically rigorous research with conclusions that are appropriately drawn based on the data presented.

Reviewer #1: Yes

**Response:** Thank you

2. Has the statistical analysis been performed appropriately and rigorously?

Reviewer #1: Yes

**Response:** Thank you

3. Have the authors made all data underlying the findings in their manuscript fully available (please refer to the Data Availability Statement at the start of the manuscript PDF file)?

The [PLOS Data policy](https://journals.plos.org/climate/s/data-availability) requires authors to make all data underlying the findings described in their manuscript fully available without restriction, with rare exception. The data should be provided as part of the manuscript or its supporting information, or deposited to a public repository. For example, in addition to summary statistics, the data points behind means, medians and variance measures should be available. If there are restrictions on publicly sharing data—e.g. participant privacy or use of data from a third party—those must be specified.

Reviewer #1: Yes

**Response:** Thank you

4. Is the manuscript presented in an intelligible fashion and written in standard English?

PLOS Global Public Health does not copyedit accepted manuscripts, so the language in submitted articles must be clear, correct, and unambiguous. Any typographical or grammatical errors should be corrected at revision, so please note any specific errors here.

Reviewer #1: Yes

**Response:** Thank you

5. Review Comments to the Author

Please use the space provided to explain your answers to the questions above. You may also include additional comments for the author, including concerns about dual publication, research ethics, or publication ethics. (Please upload your review as an attachment if it exceeds 20,000 characters)

Reviewer #1: Dear Authors,

I read with interest this manuscript and I have some questions to pose to Authors.

1) Could you please describe in more detail what VA is? I think that general readers could not be aware of characteristics of this tool. I think it can be added to the method section.

**Response:** Thank you for this suggestion and apologies for the lack of detail in the original manuscript. We have carefully revised the method section to include more details about VA methods and additional references. On lines 110 – 113 of the revised manuscript we write:

“VA implementation involves interviewing a close relative of the deceased using a structured questionnaire in order to capture sequence of events and symptoms leading to death (40-42). The establishment of the possible cause of death is then derived using physician review or computational approaches (43-45). “

2) I cannot find Appendix A1, and thus I'm asking if a table showing how the 67 causes of death were coded in the 7 categories pointed out in the manuscript has been included. If not, I think that it might be useful.

**Response:** Apologies for this – it seems like the appendix did not upload correctly in the original submission. In the revised manuscript, we appended appendix 1 at the end of the document (beginning at the line no. 535). Indeed as you have highlighted, appendix 1 contains the WHO Verbal Autopsy (VA) target list of causes of death list which are coded in the 7 categories.

3) It is not clear to me in which way the exposure in mines has been assigned. I mean, when and how long a person had to be a mine worker to be included among exposed people? I think it should be clarified and added to methods.

**Response:** We fully agree with this point. Exposure to mine was coded as a binary indicator whether a deceased person had ever worked in the mining sector or not. We have revised the method section to include this important clarification on line 140 of the revised text.

4) I think that the short follow-up period should be highlighted as a limit of this paper.

**Response:** We definitely agree that a longer surveillance period would have given us more statistical power and precision. We have added this limitation to the discussion section as suggested. Kindly see line 298-299

6. PLOS authors have the option to publish the peer review history of their article ([what does this mean?](https://journals.plos.org/climate/s/editorial-and-peer-review-process#loc-peer-review-history)). If published, this will include your full peer review and any attached files.


**Do you want your identity to be public for this peer review?** If you choose “no”, your identity will remain anonymous but your review may still be made public.


For information about this choice, including consent withdrawal, please see our [Privacy Policy](https://www.plos.org/privacy-policy).

Reviewer #1: No
