# Supplementary material for: Estimating the mortality burden of large scale mining projects—Evidence from a prospective mortality surveillance study in Tanzania
Source: PLOS Glob Public Health. 2021 Oct 13;1(10):e0000008. doi: 10.1371/journal.pgph.0000008 (PMC10021452; doi:10.1371/journal.pgph.0000008)
Supplement: Attachment — Submitted filename: Responses_to_reviewers_Sept2nd.docx [file pgph.0000008.s003.docx]

**Manuscript Number:** PGPH-D-21-00132 **Manuscript Title:** Estimating the mortality burden of large scale mining projects – Evidence from a prospective mortality surveillance study in Tanzania

**Cover letter**

Dar-es-Salaam, 1 September 2021

Dear Dr. Chirico

Thank you for your kind email from July 2^nd^, 2021. We were pleased to note the overall positive appraisal of our research and grateful for your invitation to revise and resubmit our manuscript.

We have carefully revised our manuscript in light of the reviewer’s feedback. Please find our point-by-point responses attached below. To further assist in the readily reviewing our changes, we indicate the line numbers where the revised manuscript has changed in our reply. In the revised manuscript, all changes are highlighted in yellow.

We look forward to hearing from you soon – please let us know if you have any questions or require any further information.

Yours sincerely,

Isaac Lyatuu (on behalf of all the co-authors)

**Journal specific requirements**

1. Please amend your list of authors in Editorial Manager to ensure that each author is linked to an affiliation.

Thank you. We have applied this change in the Editorial Manager. Kindly see the changes

2. Please provide separate figure files in .tif or .eps format only and remove any figures embedded in your manuscript file.  Please ensure that all files are under our size limit of 20MB.  If you are using LaTeX, you do not need to remove embedded figures

Thank you. We have removed all the figures and provided them as separate files. We further indicated where do these figures go in our main document. Pease see line 103, 211 and 219

3. In the online submission form, you indicated that "The data presented in this study are available on request from the Ministry of Health
Community Development, Gender, Elderly and Children (MoHCDGEC). The data are not publicly available due to local restrictions."

All PLOS journals now require all data underlying the findings described in their manuscript to be freely available to other researchers, either 1. In a public repository, 2. Within the manuscript itself, or 3. Uploaded as supplementary information.
This policy applies to all data except where public deposition would breach compliance with the protocol approved by your research ethics board. If your data cannot be made publicly available for ethical or legal reasons (e.g., public availability would compromise patient privacy), please explain your reasons by return email and your exemption request will be escalated to the editor for approval. Your exemption request will be handled independently and will not hold up the peer review process, but will need to be resolved should your manuscript be accepted for publication. One of the Editorial team will then be in touch if there are any issues.

Thank you for this additional clarification. While some of the data used in our manuscript is publicly available from online public repositories, some (the mortality data) is not. This data is available upon request to the ministry of health in Tanzania. Making this data publicly available would breach compliance with the approved protocol by the local research ethics board. We have added this important information in the main manuscript document at line 345-351

4. Please provide us with a direct link to the base layer of the map used in Figure 1 and ensure this location is also included in the figure legend.

Thank you. We have provided an additional text file “BaseLayerProperties.txt” which contains additional details about the base layer used in Figure 1. In additional, we have expanded the caption of figure 1 to contain more details. Please see line 104-109

**Point by point responses to reviewers**

**Manuscript Number:** PGPH-D-21-00132 **Manuscript Title:** Estimating the mortality burden of large-scale mining projects – Evidence from a prospective mortality surveillance study in Tanzania

1. Does this manuscript meet PLOS Global Public Health's [publication criteria](https://journals.plos.org/climate/s/criteria-for-publication)? Is the manuscript technically sound, and do the data support the conclusions? The manuscript must describe methodologically and ethically rigorous research with conclusions that are appropriately drawn based on the data presented.

Reviewer #1: Yes

**Response:** Thank you

2. Has the statistical analysis been performed appropriately and rigorously?

Reviewer #1: Yes

**Response:** Thank you

3. Have the authors made all data underlying the findings in their manuscript fully available (please refer to the Data Availability Statement at the start of the manuscript PDF file)?

The [PLOS Data policy](https://journals.plos.org/climate/s/data-availability) requires authors to make all data underlying the findings described in their manuscript fully available without restriction, with rare exception. The data should be provided as part of the manuscript or its supporting information, or deposited to a public repository. For example, in addition to summary statistics, the data points behind means, medians and variance measures should be available. If there are restrictions on publicly sharing data—e.g. participant privacy or use of data from a third party—those must be specified.

Reviewer #1: Yes

**Response:** Thank you

4. Is the manuscript presented in an intelligible fashion and written in standard English?

PLOS Global Public Health does not copyedit accepted manuscripts, so the language in submitted articles must be clear, correct, and unambiguous. Any typographical or grammatical errors should be corrected at revision, so please note any specific errors here.

Reviewer #1: Yes

**Response:** Thank you

5. Review Comments to the Author

Please use the space provided to explain your answers to the questions above. You may also include additional comments for the author, including concerns about dual publication, research ethics, or publication ethics. (Please upload your review as an attachment if it exceeds 20,000 characters)

Reviewer #1: Dear Authors,

I read with interest this manuscript and I have some questions to pose to Authors.

1) Could you please describe in more detail what VA is? I think that general readers could not be aware of characteristics of this tool. I think it can be added to the method section.

**Response:** Thank you for this suggestion and apologies for the lack of detail in the original manuscript. We have carefully revised the method section to include more details about VA methods and additional references. On lines 114 – 117 of the revised manuscript we write:

“VA implementation involves interviewing a close relative of the deceased using a structured questionnaire in order to capture sequence of events and symptoms leading to death (40-42). The establishment of the possible cause of death is then derived using physician review or computational approaches (43-45). “

2) I cannot find Appendix A1, and thus I'm asking if a table showing how the 67 causes of death were coded in the 7 categories pointed out in the manuscript has been included. If not, I think that it might be useful.

**Response:** Apologies for this – it seems like the appendix did not upload correctly in the original submission. In the revised manuscript, we appended appendix 1 at the end of the document (beginning at the line no. 550). Indeed as you have highlighted, appendix 1 contains the WHO Verbal Autopsy (VA) target list of causes of death list which are coded in the 7 categories.

3) It is not clear to me in which way the exposure in mines has been assigned. I mean, when and how long a person had to be a mine worker to be included among exposed people? I think it should be clarified and added to methods.

**Response:** We fully agree with this point. Exposure to mine was coded as a binary indicator whether a deceased person had ever worked in the mining sector or not. We have revised the method section to include this important clarification on line 143-144 of the revised text.

4) I think that the short follow-up period should be highlighted as a limit of this paper.

**Response:** We definitely agree that a longer surveillance period would have given us more statistical power and precision. We have added this limitation to the discussion section as suggested. Kindly see line 306-307

6. PLOS authors have the option to publish the peer review history of their article ([what does this mean?](https://journals.plos.org/climate/s/editorial-and-peer-review-process#loc-peer-review-history)). If published, this will include your full peer review and any attached files.


**Do you want your identity to be public for this peer review?** If you choose “no”, your identity will remain anonymous but your review may still be made public.


For information about this choice, including consent withdrawal, please see our [Privacy Policy](https://www.plos.org/privacy-policy).

Reviewer #1: No
